# DEEP‐DISORDER: Motion Correction in 3D MRI via Segment Reconstruction and Registration

**DOI:** 10.1002/nbm.70286

**Published:** 2026-04-09

**Authors:** Laurens Beljaards, Martijn Nagtegaal, Chinmay Rao, Yiming Dong, Matthias J. P. van Osch, Nicola Pezzotti, Mariya Doneva, Marius Staring

**Affiliations:** ^1^ Department of Radiology Leiden University Medical Center Leiden the Netherlands; ^2^ Department of Mathematics and Computer Science Eindhoven University of Technology Eindhoven the Netherlands; ^3^ Cardiologs Paris France; ^4^ Philips Innovative Technologies Hamburg Germany

**Keywords:** deep learning, DISORDER, groupwise registration, image reconstruction, retrospective motion correction

## Abstract

3D MR image acquisition is inherently time intensive, rendering it susceptible to patient motion during scanning. This may introduce significant blurring and artifacts, potentially necessitating reacquisition. We propose a modular framework to retrospectively correct for intrascan motion in 3D brain MRI, without active motion tracking. Serving as the backbone of our approach is an existing distributed and incoherent sampling scheme (DISORDER), combined with a fast network trained for highly undersampled reconstruction. This enables approximate reconstructions of anatomy after every few seconds, using only a tiny fraction of k‐space data (< 2%). While these reconstructions are only approximate, we postulate they are sufficient to estimate motion patterns at said temporal resolution. Groupwise registration, notable for its elimination of registration bias, is utilized for estimating rigid motion parameters, which are leveraged to reconstruct the measured data with reduced motion artifacts. The approach was evaluated on 94 retrospectively and 3 prospectively motion‐corrupted in vivo 3D T1‐weighted brain MRI acquisitions. The estimated motion parameters matched the known retrospective motion with 0.06 mm and 0.13° accuracy, resulting in an improvement in reconstruction quality from 0.942±0.026 to 0.992±0.003 SSIM for the retrospective scans. The prospective scans improved from 0.915±0.024 to 0.936±0.014SSIM after correction in the case of gradual motion and from 0.764±0.008 to 0.923±0.011 SSIM for extreme motion. In conclusion, the proposed approach, that is free of external tracking devices or navigators, successfully estimated and corrected 3D motion between small subportions of a scan. This resulted in vastly improved image quality, making volumetric MRI substantially more tolerant to motion.

## Graphical abstract

This work presents a retrospective motion correction framework for 3D MRI. A motion‐corrupted acquisition is split into tiny k‐space segments, and for each a neural network reconstructs a rough anatomical image. These reconstructions are aligned using groupwise registration, yielding one set of estimated motion parameters per segment. The motion parameters are used to correct for the motion in the measured data, producing an MR image with reduced motion artifacts.

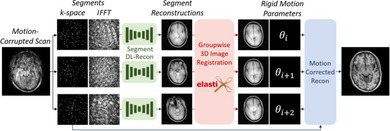

**Abbreviations**
CG: conjugate gradient
CNN: convolutional neural network
DISORDER: distributed and incoherent sample orders for reconstruction deblurring using encoding redundancy
DL‐Recon: deep learning–based reconstruction model
GAN: generative adversarial network
PSNR: peak signal‐to‐noise ratio
SENSE: sensitivity encoding
SSIM: structural similarity index measure
TFE: turbo field echo

## 1 Introduction

Multishot MRI is an imaging technique used to obtain high‐resolution anatomical images by acquiring k‐space data over multiple excitation pulses, which is a time‐intensive process that requires the subject to lie still for several minutes. This can be especially challenging for young or vulnerable patients. If the patient does move during MRI acquisition, this can introduce artifacts and distortions into the image with their severity depending on the amount of motion and may in the worst case require the scan to be reacquired. One study estimated the costs of motion at $115,000 per scanner per year in 2015 [1], while another study in 2020 estimated the annual cost of patient head motion at $45,066 per scanner without pediatric examinations and $364,242 with pediatric examinations [2]. Thus, development of approaches that make MRI more robust to motion without increasing scan time or decreasing image quality is paramount. Retrospective intrascan motion correction aims to reduce motion artifacts in the reconstruction of an MRI image, often requiring an estimate of the motion of the subject to do so [3, 4, 5, 6, 7, 8, 9]. One way of obtaining motion parameters is via tracking devices, though depending on the approach, there may be additional effort that complicates the workflow [4], increased cost, and also the accuracy of the external tracking devices may be insufficient. On the other hand, data‐driven approaches are preferred as they do not require external hardware.

Retrospective motion correction is deeply intertwined with image reconstruction, thus addressing these two problems jointly confers synergistic benefits. After all, a better motion estimate can enable better reconstruction and vice versa. Such an approach may involve incorporating a motion term in the forward model and iteratively estimating the motion parameters and motion‐corrected image [10]. An example of such a work is the DISORDER [11] framework, which additionally introduces a specialized sampling scheme. This distributed and incoherent sampling scheme maximizes the sensitivity to motion, which improves the ability to detect motion and solve the aligned reconstruction problem. While these approaches have the benefit of being data consistent, the iterative process can be computationally expensive and difficult to converge. One work proposed integrating a CNN trained to remove motion artifacts to improve the conditioning and convergence of the joint search [12]. Some approaches, on the other hand, avoid the repeated image updates in the joint optimization, for example, using a scout scan to replace the image estimate, thereby speeding up the reconstruction but slowing down the acquisition [13], or a deep learning–based reconstruction to replace the image estimate [14], optimizing only the motion parameters using a data consistency loss.

AI‐based reconstruction end‐to‐end models have fast inference times and can be trained to reconstruct images that appear free of motion artifacts, often using a physics‐informed motion‐corruption simulation to generate paired training data [5]. Approaches that do not explicitly model the motion parameters have been proposed using 3D CNNs [15], foundation models [16], Fourier aggregation [17], and GANs [18]. However, if the underlying motion is not modeled explicitly, deviating from the measured k‐space data (data inconsistency) is required to correct the motion artifacts. In the case of severe motion, such a model may be prone to hallucinations, potentially resulting in missing anatomical details or the generation of nonexistent anatomy in the reconstruction [14, 19].

We therefore propose a framework that retrospectively corrects for motion in 3D MRI requiring no external tracking device, reference scan, or fully sampled center of k‐space. The proposed approach generates reconstructions of small scan segments that are registered in a groupwise fashion, thereby estimating a time series of rigid motion parameters to be used in a final motion‐corrected reconstruction. Only the estimated motion parameters are used in the final motion‐corrected reconstruction, enabling the enforcement of data consistency and avoiding hallucinations. The estimation of motion parameters by registering some form of low‐quality reconstructions for each scan segment has been applied before in self‐navigated approaches, for example, by repeatedly sampling rotated blocks of k‐space and using the overlapping central data from each to generate low‐resolution images [20] or by apodization of the repeatedly sampled central k‐space data with a Fermi filter [21]. However, these approaches rely on a fully sampled center of k‐space for each segment, requiring a slower sequence [4] or a sequence that already oversamples the k‐space center [21]. We instead postulate that deep learning enables the reconstruction of small scan segments without a fully sampled k‐space center at a sufficient quality for motion estimation. To ensure each scan segment contains a few lines from the center of k‐space, we use the distributed incoherent sampling introduced for the DISORDER [11] method. We have therefore named our framework DEEP‐DISORDER.

In the following, we detail our approach, and compare it to the original DISORDER reconstruction on retrospectively motion‐corrupted data, as well as on prospectively motion‐corrupted and motion‐free 3D T1‐weighted in vivo brain scans.

## 2 Methods

### 2.1 Problem Definition

The data acquisition process is modeled by the forward equation y=Ax, with y denoting the measured k‐space data, x denoting the underlying image, and the forward operator $A=MFSC_{\theta }$ describing the acquisition process. Here, $C_{\theta }$ is the segment‐wise motion‐corruption operator that projects the image into different motion states with different motion parameters θ. In the proposed pipeline, trilinear interpolation is used in the operator $C_{\theta }$. S represents the coil sensitivity encoding operator, F is the Fourier transform, and M is the masking operator that selects k‐space lines by applying different sampling masks to the different segments. We define a segment as a fraction of k‐space that represents one estimated motion state. As our acquisition process (a 3D‐T1‐weighted TFE sequence) comprises multiple shots, it follows naturally that we partition the k‐space at shot boundaries with each segment consisting of a whole number of shots. Estimated motion parameters $\hat{\theta }$ can be used to invert the displacements and rotations that occurred during the acquisition for each segment, yielding the motion‐corrected reconstruction problem

(1) $$\hat{x}=\underset{x}{\text{argmin}}\|MFSC_{\hat{\theta }}x-y{\|}_{2}^{2},$$

which can be solved using CG‐SENSE [22] adapted for motion compensation [18, 23]. Given the estimated forward operator $A=MFSC_{\hat{\theta }}$, the least‐squares solution for a given A can be found by solving the equation:

(2) $$A^{H}Ax=A^{H}y.$$

Because the matrix $A^{H}A$ is too large to be inverted or stored explicitly, the system is solved iteratively using the conjugate gradient (CG) algorithm [24].

### 2.2 DISORDER Reconstruction

The original DISORDER reconstruction approach [11] solves for x by iteratively updating the estimated motion parameters θ for a fixed image and updating the image using fixed motion parameters:

(3) $${\hat{x}}^{\left(i+1\right)}=\underset{x}{\text{argmin}}\|MFSC_{{\hat{\theta }}^{\left(i\right)}}x-y{\|}_{2}^{2},$$

(4) $${\hat{\theta }}^{\left(i+1\right)}=\underset{\theta }{\text{argmin}}\|MFSC_{\theta }{\hat{x}}^{\left(i+1\right)}-y{\|}_{2}^{2}.$$

DISORDER introduced a distributed incoherent sampling scheme that for each shot acquires a broad variety of frequencies from all over k‐space, making the motion easier to detect and making the problem easier to solve. We feed the final motion parameters estimated by DISORDER to the same motion‐corrected CG‐SENSE algorithm described in Equation (1) to obtain a motion‐corrected reconstruction comparable with the proposed approach.

### 2.3 DEEP‐DISORDER Reconstruction

**FIGURE 1:**
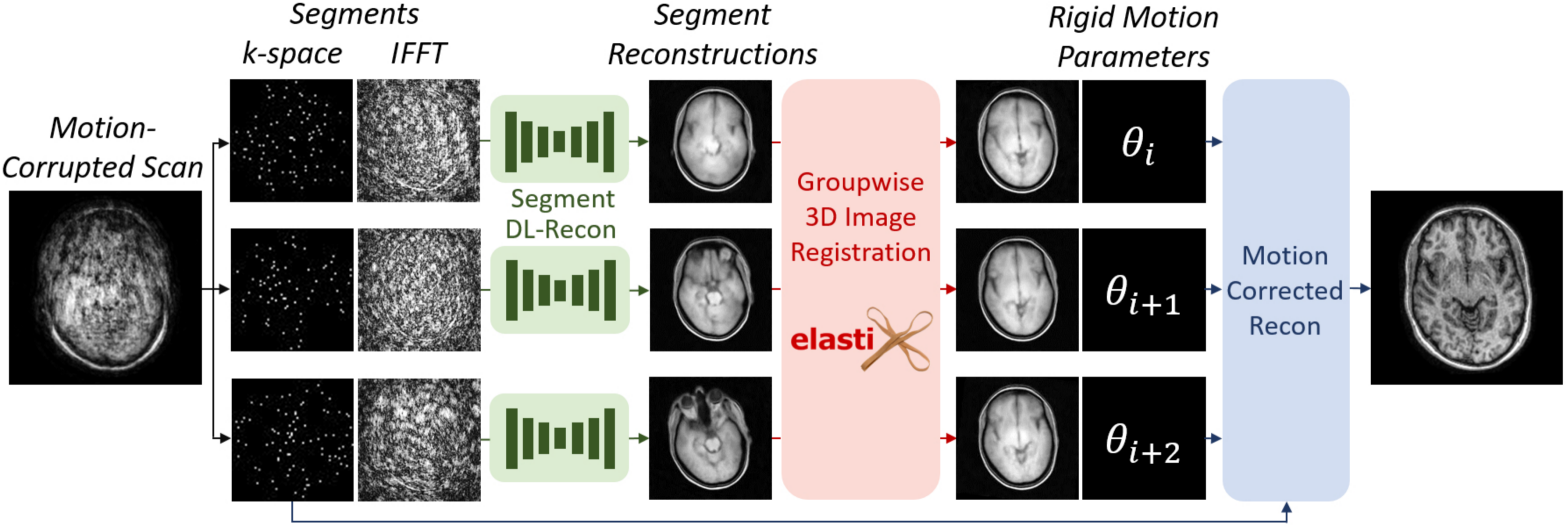
Overview of the proposed motion correction approach. The acquired motion‐corrupted k‐space is split into segments based on acquisition time. A zero‐filled fast Fourier transform is applied and an unrolled 3D U‐Net performs reconstructions for each extremely undersampled segment. These reconstructions are aligned using groupwise registration, yielding one set of estimated motion parameters per segment. Finally, these motion parameters and the measured data are used in the forward operator of a classical reconstruction approach to produce a motion‐corrected image. The coil sensitivities are omitted from the figure for clarity.

An overview of the proposed retrospectively motion‐corrected MRI reconstruction approach is given in Figure 1. The framework estimates 3D motion by reconstructing and registering segments of the acquired MRI data. We use the DISORDER sampling scheme to ensure a balanced distribution of low and high frequencies in every shot. As a single shot may contain very little anatomical information, we group multiple shots into a segment to reach an image quality that is sufficient for the subsequent registration process. Reconstructing the segments by zero filling does not yield sufficient image quality for registration as these reconstructions lack clear anatomical features due to the extremely high acceleration factor, and each segment has different undersampling artifacts, as seen in Figure 1. Moreover, the segment reconstruction problem is too ill‐posed for classical reconstruction algorithms. Therefore, we pass the segments through a deep learning reconstruction network that was trained to reconstruct extremely highly undersampled data. The segment reconstruction network $f_{\text{recon}}$ reconstructs the nth k‐space segment as

(5) $${\hat{x}}_{n}=f_{\text{recon}}\left(S^{H}F^{-1}M_{n}^{H}y;\phi \right).$$

During training, a random segment index r and random rigid transformation $C_{r}$ are generated to obtain a synthetic motion‐corrupted k‐space segment $y_{r}=M_{r}FSC_{r}x$ from uncorrupted image x. The network parameters ϕ are learned according to the optimization objective

(6) $$\phi^{*}=\underset{\phi }{\mathrm{argmin}}ℒ\left(C_{r}x,f_{\text{recon}}(S^{H}F^{-1}M_{r}^{H}y_{r};\phi )\right)$$

with loss function ℒ. These segment reconstructions are limited in quality due to the high undersampling factor and resulting lack of information, but we postulate that they are sufficiently accurate for registration purposes. The magnitude images of the segment reconstructions are registered with groupwise registration [25] using the medical image registration toolbox Elastix [26, 27]. By employing groupwise registration, we avoid having to select an arbitrary fixed image, eliminating bias in the selection by optimizing the group objective across all images via a consensus template representative of the entire group. This could be especially relevant if the quality of the segment reconstructions varies, which could potentially happen in the case of intrasegment motion, or variations in coil sensitivity due to the position of the subject. Additionally, groupwise registration avoids the accumulation of errors inherent in sequential pairwise registrations because transformations are jointly estimated.

A registration mask was used to ignore the neck region, which is often affected by nonrigid motion and instead focus on head registration. During inference, the segment reconstructions are registered to estimate the rigid motion parameters

(7) $$\{{\hat{\theta }}_{1},\text{…},{\hat{\theta }}_{N}\}=f_{\text{reg}}(\{{\hat{x}}_{1},\text{…},{\hat{x}}_{N}\}).$$

The groupwise registration objective function of $f_{\text{reg}}$ is described by

(8) $$\{\hat{\theta },\hat{T}\}=\underset{\theta ,\;T}{\mathrm{argmin}}\;\overset{N}{\underset{s=1}{\sum }}\left\{\underset{v}{\sum }{\left\|\theta_{s}\left({\hat{x}}_{s}(v)\right)-T(v)\right\|}_{2}^{2}+\mathrm{dist}\left(e,\;\theta_{s}\right)\right\}.$$

Here, $\theta_{s}$ is the transformation for segment s, T(v) is the common template for voxel v, and $\mathrm{dist}(e,\;\theta_{s})$ is a regularization term enforcing closeness to the identity transformation e [28].

Finally, as described in Equation (1), the resulting registration parameters are used as motion parameters in the motion‐corrected CG‐SENSE reconstruction algorithm to reconstruct the measured data with greatly reduced motion artifacts.

### 2.4 Training and Implementation Details

For segment reconstruction, we trained an unrolled 3D U‐Net [29, 30] with four resolution levels with 16, 32, 64, and 128 feature maps per level. Each layer of the model consists of group normalization, 3D convolution, and ReLU operators, respectively. The model received a highly undersampled zero‐filled 3D image as input and was tasked to estimate the corresponding uncorrupted fully sampled 3D image. A blend of SSIM [31] loss and smooth‐L1 loss was used during training. Both the input and target were randomly rotated and translated during training to account for different patient positions.

Unrolled or iterative networks are commonly used in MRI reconstruction [32, 33], as they enable iterative reconstruction in which data consistency is continually enforced. Because each segment has few k‐space lines, the benefit of data consistency that an iterative approach provides is small. Nonetheless, we observed a minor improvement in performance over a single‐stage approach from 0.043 to 0.033 NMSE on 90 retrospectively corrupted scans, albeit at the cost of an increased GPU memory footprint. We thus use a segment reconstruction approach with six consecutive residual U‐Nets that were trained end to end. The reconstruction models were pretrained for 16 segments on an NVIDIA RTX A6000 GPU for $1\times 10^{6}$ iterations with a batch size of 2 and a group normalization size of 8 and fine‐tuned on the desired number of segments for $4\times 10^{5}$ iterations, which was 64 for most experiments. We used a leave‐one‐out approach to reduce out‐of‐distribution issues on the test set, by further fine‐tuning multiple models on a subset of motion‐free volunteer scans and evaluating each on the unseen volunteer.

For groupwise registration, we used Elastix with a multiresolution pyramid of four levels and 2500 iterations per resolution. The used registration pipeline and settings differ from DISORDER, as the proposed approach performs only a final groupwise registration while DISORDER performs many quick motion update steps alternating with reconstruction steps. We selected a high number of registration iterations and three repetitions of the registration schedule to ensure convergence. We did not optimize these settings for runtime performance. The registration took about 10 min for R=2 on the CPU.

### 2.5 Number of Segments

**FIGURE 2:**
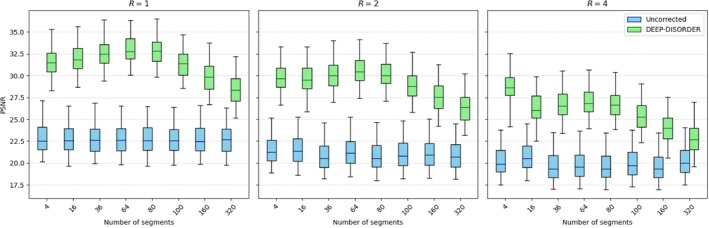
Motion‐corrected reconstruction performance on synthetic data with different numbers of shots, with one shot per segment.

Besides the hyperparameters of the segment reconstruction and registration, there are only a few factors of influence for our method, most notably the number of segments. There is a natural tradeoff between the achievable image quality for each segment and the temporal resolution for the motion correction. To find a balanced configuration for which the proposed approach performs best, we trained and evaluated our approach on acquisition patterns with different numbers of segments, which were constructed using random‐checkered sampling [11]. Figure 2 shows the reconstruction performance for a given number of segments on 90 retrospectively sampled motion‐corrupted scans with shaking or nodding‐like motion with 320 motion states, demonstrating that the ideal number of segments is around 64 for our current setup.

### 2.6 Generalizability

An experiment is included in Appendix S1 that investigates generalizability to different retrospectively motion‐corrupted data, specifically two spoiled gradient echo 3D scans with different echo times, with and without fine‐tuning. The segment reconstruction model can handle unseen data that are moderately similar to the training data, but the performance lowers as the contrast gets further from T1w data which the model was trained on. It is demonstrated that this issue can be solved by including a number of scans of the unseen contrast in the training set. Additionally, successful correction of motion in higher resolution scans ($384^{3}$ voxels) was demonstrated when the motion estimation is run on a lower resolution ($192^{3}$ voxels) to reduce the need for compute resources.

### 2.7 Data

#### 2.7.1 Retrospectively Motion‐Corrupted Data

We focus our experiments on T1‐weighted 3D MRI scans. We used an in‐house dataset of 361 3D T1‐weighted turbo field echo scans from our radiological clinic without intentional motion for training and validation and 94 for the synthetic test set. Four hundred sixteen of the scans were acquired on Philips Ingenia 3.0T scanners and 39 on an Ingenia Elition 3.0T X scanner. All participants provided informed consent, and the study was approved by the institutional review board. The scans were performed in a transverse orientation. The flip angle was 8°, the repetition time (TR) around 9.9 ms and the echo time (TE) around 4.6 ms. The scans were acquired with 1.8× acceleration. Coil‐combined images were obtained using the scanner's built‐in compressed sensing reconstruction without postprocessing. These scans were retrospectively motion corrupted by simulating the k‐space acquisition process, giving us paired motion‐corrupted and ground‐truth data. For each motion state, we applied a translation and rotation to the coil‐combined image, and we sampled k‐space lines from this transformed image according to the acquisition pattern. The acquisition matrices of the original data had different sizes, which we cropped or zero padded to $240^{3}$. For intersegment motion, we used one motion state per segment, meaning 64 motion states with multiple shots sharing one motion state. This is less realistic but matches the number of motion states our approach can estimate, allowing us to see how well it resolves correctable motion. To simulate intrasegment motion, we used 320 motion states, giving each shot its own motion state. We used a TFE factor of 180 in our simulated acquisitions, meaning 180 k‐space lines per shot and 320 shots, resulting in 900 k‐space lines per segment for 64 segments. We obtained coil sensitivity maps from the SENSE reference scan that is automatically acquired for sequences that require coil sensitivity maps for image reconstruction. The coil sensitivity maps were compressed to eight coils by a singular value decomposition [34] and normalized using root‐sum‐of‐squares normalization. The scans were retrospectively undersampled by discarding a number of shots corresponding to the desired acceleration factor. Different motion patterns were used in the different experiments. To create random intersegment motion, we sampled 64 translations and rotations from a normal distribution with a standard deviation of 2 mm and 1° for each axis (“spatially medium motion”) or 12 mm and 6° (“spatially extreme motion”). This resulted in an average distance between the centers of two segments of 4.5 or 27.1 mm, respectively. We optionally added intrasegment motion on top of this motion, randomly generated in a similar fashion but with 320 motion states and only 1/16th as severe, as we expected such motion to be less severe due to the shorter timeframe. Besides the random motion patterns, we also used more realistic rigid head motion patterns from a previous work [35] where a scanner with an optical tracking system was used to track participants who were instructed to perform shaking or nodding‐like motion.

#### 2.7.2 Prospectively Motion‐Corrupted Data

We acquired additional 3D T1‐weighted prospectively motion‐corrupted scans and still reference scans from three healthy volunteers for testing and one for validation, who all provided informed consent. The volunteers were instructed to move according to a specified motion pattern in each scan. For the gradual motion pattern, the volunteers were asked to occasionally slightly rotate their head as if slowly looking around, mimicking the drift often seen in clinical settings by patients who gradually relax or tire. For the jittery motion pattern, the volunteers were asked to abruptly rotate their head in a random direction approximately once every 15 s, such that their nose moves by approximately 2 cm each time. For the spatially extreme motion pattern, the volunteers were asked to look as far as possible to the bottom left at the start of the acquisition and rotate their head until they faced the top right at the end of the acquisition. The volunteers were informed in advance of the length of the scan and were asked to perform this motion in a stepwise fashion by moving every 15 s as it can be straining to slowly move continuously. This results in a reproducible large motion pattern with visible progression, which enables visually checking the reconstructed segments and registration for correctness while no ground‐truth motion is available. The first, middle, and last segment reconstructions of the volunteer used for validation can be seen in Figure 1. We did not apply padding in the head coil, to enable more severe motion and test the limits of what motion can be corrected. The acquisitions used the MP‐RAGE [36] sequence and were acquired on a 1.5T scanner (Ingenia, Philips Healthcare, Best, the Netherlands). The acquisitions were of size 191×191×384 and consisted of 133 or 68 shots for prospective accelerations R=1 and R=2 with a TFE factor of 216. Using three shots per segment, this leads to 648 k‐space lines per segment. We used flip angles of 8°, TR of 4.6 ms, and TE of 2.1 ms. The acquisitions lasted 6 min and 44 s for R=1 and 3 min and 29 s for R=2 and were performed in a sagittal orientation. Coil sensitivity maps were again obtained from the automatically acquired SENSE reference scan, compressed to eight coils and normalized.

## 3 Results

For evaluation, we compare our method against DISORDER, which is closest to our work and an excellent example of another navigator‐free and retrospective approach. In Section 3.1, we evaluate both the DISORDER and proposed DEEP‐DISORDER approach on retrospectively motion‐corrupted data. Section 3.2 shows the performance of both approaches on deliberately prospectively motion‐corrupted MRI acquisitions of healthy volunteers. We selected the segment size to be three shots for both approaches on the retrospectively corrupted data. The final reconstructions were obtained by using the estimated motion parameters in the exact same motion‐corrected CG‐SENSE reconstruction algorithm with the same settings for both DISORDER and DEEP‐DISORDER. Thus, the only difference in reconstruction quality between the approaches comes from the estimated motion. As is well known [37], running the CG algorithm for too many iterations can lead to amplified noise in the final reconstruction, so we selected 10 and 3 iterations for the retrospectively and prospectively corrupted data, respectively. The reconstruction quality was evaluated using two measures: the structural similarity index measure (SSIM) [31], which quantifies perceptual similarity by comparing luminance, contrast, and structural information, and the peak signal‐to‐noise ratio (PSNR), which quantifies reconstruction fidelity via the ratio of the maximum possible signal power to the error power.

### 3.1 Evaluation on Retrospectively Motion‐Corrupted Data

**FIGURE 3:**
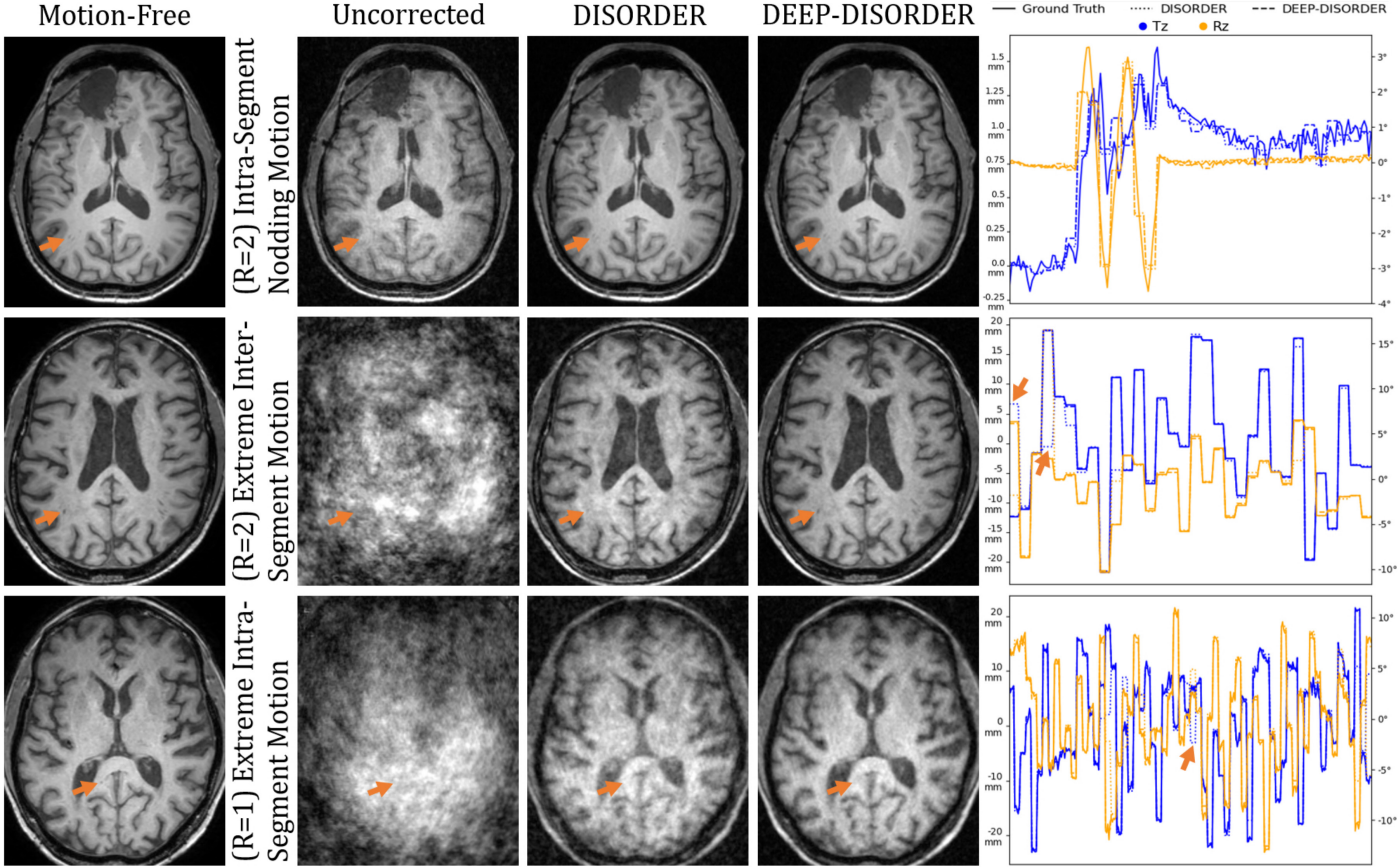
Reconstructions of retrospectively motion‐corrupted and retrospectively undersampled data. The arrows highlight tiny structures that are rendered imperceptible by motion artifacts. DISORDER and DEEP‐DISORDER correct the majority of motion artifacts, making the structures visible again for all but the most extreme motion patterns. The estimated and ground‐truth motion parameters are shown for a selected axis of translation (T) and rotation (R), with arrows highlighting outlying estimations by DISORDER.

Table 1 shows a comparison on the retrospectively motion‐corrupted test set between a baseline uncorrected approach that uses a zero‐motion estimate, DISORDER and DEEP‐DISORDER. Each approach estimated 64 motion states. The first row evaluates DISORDER and DEEP‐DISORDER on motion‐free data. DISORDER estimates motion closer to zero due to the nature of its optimization loop, but neither approach deteriorates image quality. For medium random motion and nodding motion, both approaches correct the motion similarly well enough to produce near‐perfect reconstructions. Of these, the pattern with the lowest SSIM after correction (R=2 intrasegment nodding motion) is shown in the top row of Figure 3. The motion‐corrected image is almost indistinguishable from the motion‐free image, bar a slight increase in noise. The remaining images show the performance for extreme motion. Once again, a major improvement by both approaches over the uncorrected image can be seen, but unlike before, DEEP‐DISORDER corrects it more accurately, both in quantitative measures and in visual appearance. For the extreme intrasegment motion, neither approach sufficiently corrects for the motion. After all, the motion that happens within a segment is not estimated and therefore uncorrected. While this extreme motion pattern effectively illustrates the breaking point of both approaches, the displacements are too large to typically occur in practice. In practice, the breaking point may be at less severe motion because not all effects of motion can be accounted for in the forward model.

**TABLE 1:** Mean and standard deviation of the motion‐corrected reconstruction quality on the retrospectively motion‐corrupted test set of 94 subjects.

Simulated	Motion estimation	Motion‐corrected reconstruction SSIM (μ±σ)			Mean motion error		Segment recon
Motion Pattern		R=1	R=2	R=4	mm	°	SSIM
Still	None	1	0.999 ± 0.000	0.968 ± 0.005	—	—	—

DISORDER	1.000 ± 0.000	0.999 ± 0.000	0.968 ± 0.005	0.01 ± 0.00	0.01 ± 0.00	—

DEEP‐DISORDER	0.999 ± 0.000	0.998 ± 0.001	0.967 ± 0.006	0.05 ± 0.01	0.09 ± 0.01	0.823 ± 0.023
Random	None	0.791 ± 0.021	0.768 ± 0.024	0.743 ± 0.026	—	—	—
intersegment	DISORDER	1.000 ± 0.000^a^	0.996 ± 0.001^a^	0.957 ± 0.006^a^	0.03 ± 0.01	0.06 ± 0.02	—
	DEEP‐DISORDER	0.999 ± 0.000^a^	0.995 ± 0.001^a^	0.955 ± 0.007^a^	0.06 ± 0.01	0.13 ± 0.02	0.839 ± 0.021
Random	None	0.790 ± 0.022	0.768 ± 0.024	0.744 ± 0.027	—	—	—
intrasegment	DISORDER	0.997 ± 0.000^a^	0.991 ± 0.001^a^	0.951 ± 0.007^a^	0.21 ± 0.01	0.12 ± 0.01	—
	DEEP‐DISORDER	0.996 ± 0.001^a^	0.990 ± 0.002^a^	0.950 ± 0.007^a^	0.20 ± 0.01	0.16 ± 0.02	0.838 ± 0.021
Nodding	None	0.941 ± 0.026	0.915 ± 0.035	0.844 ± 0.054	—	—	—
from tracking device	DISORDER	1.000 ± 0.000^a^	0.997 ± 0.001^a^	0.959 ± 0.007^a^	0.04 ± 0.01	0.07 ± 0.02	—
intersegment	DEEP‐DISORDER	0.999 ± 0.000^a^	0.996 ± 0.001^a^	0.957 ± 0.008^a^	0.06 ± 0.01	0.13 ± 0.02	0.838 ± 0.021
Nodding	None	0.942 ± 0.026	0.917 ± 0.035	0.847 ± 0.054	—	—	—
from tracking device	DISORDER	0.993 ± 0.003^a^	0.987 ± 0.006^a^	0.940 ± 0.017^a^	0.25 ± 0.10	0.42 ± 0.11	—
intrasegment	DEEP‐DISORDER	0.992 ± 0.003^a^	0.986 ± 0.006^a^	0.938 ± 0.018^a^	0.25 ± 0.09	0.44 ± 0.11	0.834 ± 0.022
Spatially extreme	None	0.708 ± 0.025	0.697 ± 0.028	0.684 ± 0.030	—	—	—
random	DISORDER	0.945 ± 0.020^a^	0.918 ± 0.029^a^	0.873 ± 0.032^a^	7.14 ± 2.01	2.32 ± 0.81	—
intersegment	DEEP‐DISORDER	0.992 ± 0.003^a^	0.982 ± 0.005^a^	0.942 ± 0.010^a^	0.16 ± 0.04	0.32 ± 0.05	0.832 ± 0.021
Spatially extreme	None	0.707 ± 0.025	0.697 ± 0.028	0.684 ± 0.030	—	—	—
random	DISORDER	0.893 ± 0.018^a^	0.866 ± 0.022^a^	0.827 ± 0.027^a^	7.67 ± 1.74	2.63 ± 0.80	—
intrasegment	DEEP‐DISORDER	0.927 ± 0.009^a^	0.905 ± 0.012^a^	0.865 ± 0.017^a^	1.24 ± 0.05	0.83 ± 0.08	0.803 ± 0.024

*Note:* The mean registration distance and segment reconstruction quality were calculated for R=1 as it contains all shots. The mean motion error was obtained by averaging the distance between the 64 estimated and 64 or 320 known rigid motion parameters after adding a motion that registers the corrected result to the target. The standard deviation expresses the variation between the population of 94 scans not between individual segments.

^a^ A significant improvement over the uncorrected reconstructions.

### 3.2 Evaluation on Prospectively Motion‐Corrupted Data

**FIGURE 4:**
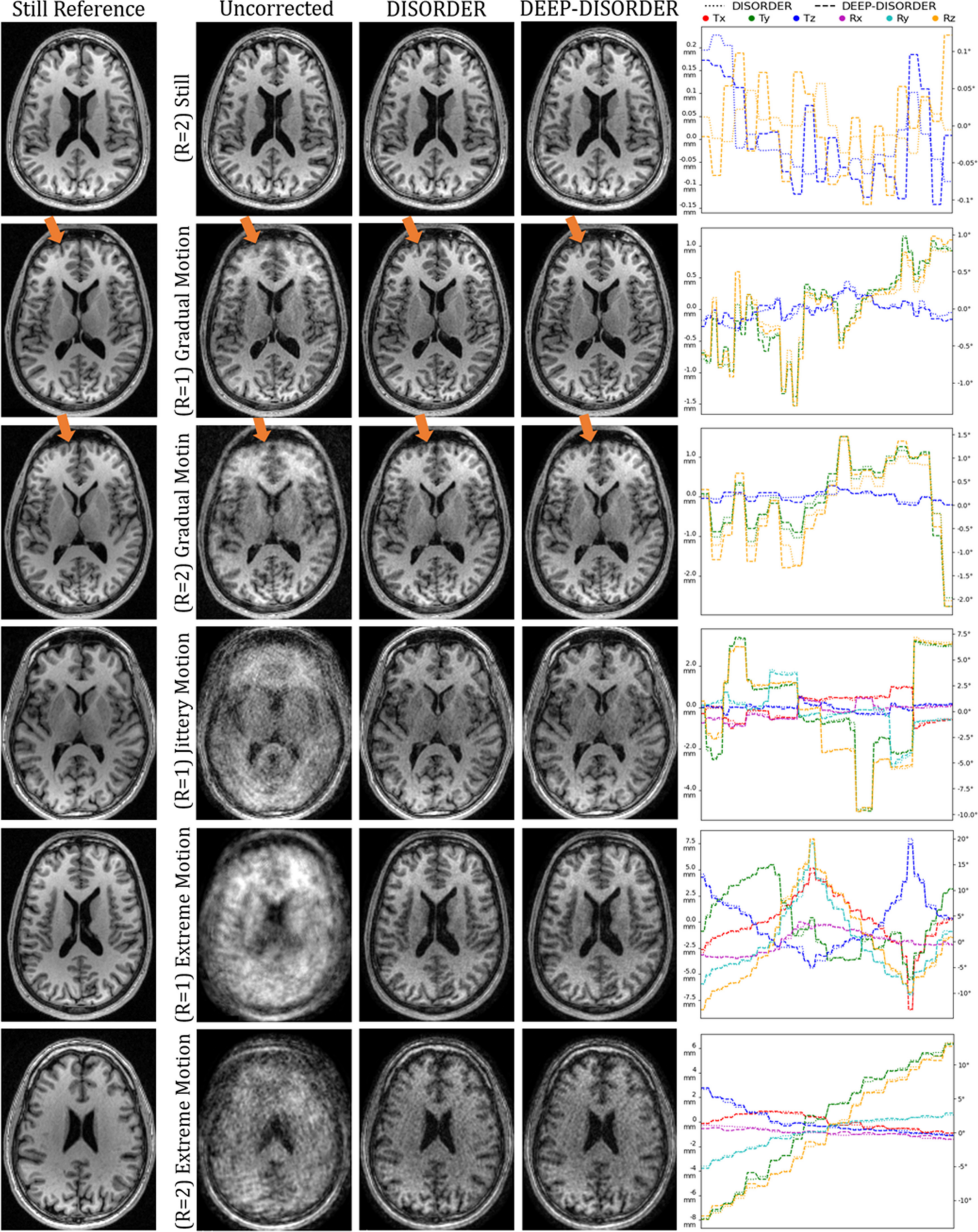
Reconstructions of prospectively motion‐corrupted data, and corresponding R=1 still reference scans. The arrows highlight blurring caused by volunteer motion, most of which is corrected by DISORDER and DEEP‐DISORDER. Arbitrarily selected translations (T) and rotations (R) are plotted on the right.

Table 2 evaluates the baseline motion‐agnostic reconstruction approach, the DISORDER approach and DEEP‐DISORDER on prospectively motion‐corrupted in vivo data. The motion‐corrected reconstructions are registered to a motion‐free reference acquisition for evaluation. The lower neck area was ignored during this registration via a mask as there may be physical deformations between the reconstructed scans and the reference. The mouth and lower neck area were masked out for quantitative evaluation because the brain is the region of interest. The first row of Figure 4 shows the reconstructions of both approaches on volunteer acquisitions without intentional motion. The higher noise level compared to the R=1 reference scan can be attributed in large part to the fact that the data is two times undersampled, as the uncorrected reconstruction also shows increased noise. The results demonstrate that both approaches do not meaningfully deteriorate image quality even in cases without motion. On scans where the volunteers performed gradual motions, we see major blurring in the uncorrected reconstructions, especially at the front of the head, as highlighted by the arrows. The values in Table 2 do not show a large increase in SSIM for the gradual motion, but the reconstructions of DISORDER and DEEP‐DISORDER are greatly deblurred as seen in the figure. Some amplified noise and minor artifacts remain visible, particularly in the R=2 case. The remaining images show examples of severe jittery and extreme motion. The uncorrected reconstructions have lost all detail, while DISORDER and DEEP‐DISORDER produce clearly improved reconstructions. The R=1 jittery and R=1 extreme motion‐corrected reconstructions no longer contain structural artifacts that hinder diagnostic ability, but the noise is highly amplified relative to the reference scan. Finally, the bottom example with R=2 extreme motion could not be sufficiently corrected and contains both structural artifacts and severe noise. Interestingly, the same artifacts can be seen here in both the DISORDER and DEEP‐DISORDER reconstructions on the left side of the brain, indicating that this motion could potentially be uncorrectable with the used number of segments.

**TABLE 2:** Mean and standard deviation of motion‐corrected reconstruction performance on prospectively motion‐corrupted volunteer scans with intentional motion.

Volunteer	Motion estimation	Motion‐corrected reconstruction quality (μ±σ)			
motion		R=1, SSIM	R=2, SSIM	R=1, PSNR	R=2, PSNR
Still	None	1	0.977 ± 0.002	∞	37.1 ± 0.1

DISORDER	0.993 ± 0.003	0.979 ± 0.001	40.8 ± 1.8	37.5 ± 0.1

DEEP‐DISORDER	0.994 ± 0.002	0.977 ± 0.001	41.7 ± 1.8	37.3 ± 0.2
Gradual	None	0.915 ± 0.024	0.901 ± 0.032	30.1 ± 1.4	29.7 ± 1.8
	DISORDER	0.937 ± 0.013	0.921 ± 0.027	31.0 ± 0.9	30.6 ± 1.7
	DEEP‐DISORDER	0.936 ± 0.014	0.920 ± 0.027	31.0 ± 1.0	30.6 ± 1.7
Jittery	None	0.837 ± 0.034	0.850 ± 0.051	27.3 ± 1.8	27.7 ± 2.3
	DISORDER	0.944 ± 0.009	0.923 ± 0.021	32.2 ± 1.0	30.8 ± 1.6
	DEEP‐DISORDER	0.941 ± 0.011	0.919 ± 0.024	31.9 ± 1.2	30.5 ± 1.6
Extreme	None	0.764 ± 0.008	0.775 ± 0.017	24.6 ± 0.7	24.9 ± 1.0
	DISORDER	0.927 ± 0.009	0.910 ± 0.016	30.9 ± 0.8	29.9 ± 0.7
	DEEP‐DISORDER	0.923 ± 0.011	0.904 ± 0.014	30.7 ± 0.9	29.6 ± 0.6

## 4 Discussion and Conclusion

We presented a framework for motion estimation and motion‐corrected reconstruction. The proposed approach reconstructs fractions (segments) of k‐space and registers them to estimate the motion of the subject. The estimated parameters can be used in a motion‐corrected reconstruction, such as a CG‐SENSE reconstruction, to remove the majority of motion artifacts.

On the retrospectively motion‐corrupted dataset, both the proposed DEEP‐DISORDER and the reference approach (DISORDER) produced visually flawless images in the case of mild intersegment motion. Neither approach perceptually degraded the image quality when no motion was present, indicating there is no downside to applying the motion correction when patients lie still. For spatially extreme intersegment motion, DISORDER saw a drop in performance while DEEP‐DISORDER successfully corrected for it with 0.16‐mm accuracy. While DISORDER implicitly aligns the segments by optimizing only for the k‐space fit, DEEP‐DISORDER performs explicit image‐domain registration. As DEEP‐DISORDER's segment reconstruction approach operates at full resolution, it can generate images with improved low‐frequency information that is preserved when downscaling the images. This enables the use of a Gaussian multiresolution pyramid strategy, which potentially increases the capture range and accuracy. A multiresolution pipeline for DISORDER on the other hand would entail cropping k‐space, reducing data to four to five k‐space lines per shot for the lowest resolution, and it is not clear if DISORDER would still work with this little information. DEEP‐DISORDER's explicit registration also enables the use of similarity metrics in the spatial domain, which potentially enriches or regularizes the problem definition, thereby making the optimization easier and more flexible. The experiments with retrospective intrasegment motion demonstrated that rapid motion occurring within a segment will elude correction, introducing mild blurring artifacts. However, in a realistic scenario, the brevity of each segment's acquisition window probably limits the severity of such intrasegment motion. Nevertheless, future research could investigate the possibility of intrasegment correction, potentially in combination with lower resolution registrations or deep learning–based registration to lower the registration time. Another direction for future work could be to explore hybrid solutions where DEEP‐DISORDER is used to initialize a DISORDER reconstruction, for example, the estimation of large motion by DEEP‐DISORDER followed by refinement of the motion parameters at finer temporal scales by DISORDER.

For the prospectively motion‐corrupted volunteer data, DISORDER and the proposed approach vastly reduced motion artifacts in the images, particularly for realistic gradual motion. The artifacts and noise amplification from the most extreme motion could not be completely corrected, though these voluntary motions were more severe than can be expected in practice as a result of our instructions to the volunteers because we wanted to find the breaking point of the correction approaches, and the fact that we deliberately scanned without padding in the head coil. The fact that the motion‐corrected reconstructions of the prospectively motion‐corrupted data still contain some motion artifacts and noise compared to the motion‐free reference scans can be attributed to multiple factors. Firstly, not all effects of motion can be accounted for in the forward model, such as the intricate effects of motion on the coil sensitivities, B0 inhomogeneities, and spin history effects. This directly hampers the final motion‐corrected reconstruction quality and potentially indirectly the segment reconstruction quality through less realistic training data. Mismatches between the coil sensitivities and the undersampled data due to motion are known to lead to aliasing and artifacts [38]. Motion also results in suboptimal k‐space sampling, thereby increasing the effective undersampling factor. Secondly, we used the basic CG solver in our motion‐corrected iterative reconstruction. While an optimized reconstruction algorithm with postprocessing or denoising may produce better images, this was not studied by us as the focus was on the motion estimation. The approach performed slightly worse on the volunteer dataset when the segment reconstruction model was not fine‐tuned on volunteer scans, mostly as a result of different acquisition settings between the test set that was sampled sagittally and the training set that was sampled transversely, negatively impacting the motion estimation. This seems to highlight a downside of our approach, as it involves training a 3D U‐Net, which requires enough training data to be available and may limit generalizability if the data is not varied. However, deep learning–based products that can reconstruct a wide variety of contrasts (T1w, T2w, FLAIR, etc.) with different acquisition settings and k‐space coverage are in the portfolio of every major scanner vendor nowadays. It stands to reason that a segment reconstruction model can similarly learn to reconstruct segments from a wide range of contrasts and different k‐space coverages if supplied with enough varied data during training. While we used 64 segments in the experiments, the optimal number of segments could vary depending on factors like the spatiotemporal scale of the acquisition, acquisition geometry, coil profiles, and levels of motion. This optimal number is however unknown at scan time, as, for example, subject motion is not known in advance. Another point of attention is that for inference the motion‐corrupted scans should be sampled with DISORDER‐like sampling, which samples the same k‐space lines as conventional sampling but in a different order to optimize the detectability of the motion.

Registered segments could be potentially checked automatically for quality issues as all segments show the same anatomy. For example, image difference metrics between the registered segment reconstructions could be used to estimate potential issues by corrupted shots within a segment. Additionally, corrupted shots at the border between two adjacent segments could be detected by checking if the absolute distance between the corresponding motion parameters in the estimated motion trajectory exceeds a certain threshold. During the final motion‐corrected reconstruction, individual shots or segments could be rejected or reduced in weight. Furthermore, data consistency metrics during the final reconstruction may help to identify outliers among the shots to reduce their influence on the final image quality. Moreover, when estimated continuously, feedback of estimated motion could help improve the efficiency of MRI examination. For example, the first two segments could already be used to detect motion early in the scan in less than 4% of total scan time, enabling the MR technician to provide feedback to the patient, or acquiring additional data in an on‐line manner when motion is detected during scanning.

In conclusion, this work outlined a framework for retrospectively correcting motion artifacts in 3D MR images. We introduced a deep learning‐based segment reconstruction model that can estimate the anatomy of in vivo subjects on a fine temporal resolution (1/64th of k‐space). These approximate reconstructions enable estimating subject motion using conventional groupwise registration. Finally, the estimated motion parameters can be leveraged in a motion‐corrected reconstruction algorithm. In an evaluation on scans from volunteers, the approach did not deteriorate motion‐free scans while removing the vast majority of motion artifacts from motion‐corrupted scans, making volumetric MRI substantially more tolerant to motion. The proposed approach is modular and flexible in nature. For example, the rigid registration component could be replaced with deformable registration to enable nonrigid motion correction, which can be challenging for conventional iterative approaches. This may be explored in future work.

## Author Contributions

**Laurens Beljaards:** conceptualization; data curation; formal analysis; investigation; methodology; software; validation; visualization; writing – original draft; writing – review and editing. **Martijn Nagtegaal:** data curation; investigation; methodology; writing – review and editing. **Chinmay Rao:** data curation; software; writing – review and editing. **Yiming Dong:** data curation; software; writing – review and editing. **Matthias J. P. van Osch:** conceptualization; funding acquisition; methodology; supervision; writing – review and editing. **Nicola Pezzotti:** conceptualization; funding acquisition; methodology; software; supervision; writing – review and editing. **Mariya Doneva:** conceptualization; methodology; supervision; writing – review and editing. **Marius Staring:** conceptualization; funding acquisition; methodology; project administration; resources; supervision; writing – review and editing.

## Conflicts of Interest

The authors declare the following financial interests or personal relationships that may be considered as potential competing interests: Laurens Beljaards and Chinmay Rao declare the grant and research support they receive from Philips. Mariya Doneva declares employment at Philips. Nicola Pezzotti declares former employment at Philips. The C.J. Gorter MRI Center receives research support from Philips. The other authors declare that they have no known competing financial interests or personal relationships that could have appeared to influence the work reported in this paper.

## Supporting information



AI4MRI_DEEPDISORDER_Appendix.pdf.

## Data Availability

The authors do not have permission to share data. Code is available at https://github.com/MoCoMRI/DEEP‐DISORDER.

## References

[1] J. B. Andre , B. W. Bresnahan , M. Mossa‐Basha , et al., “Toward Quantifying the Prevalence, Severity, and Cost Associated With Patient Motion During Clinical MR Examinations,” Journal of the American College of Radiology 12, no. 7 (2015): 689–695, 10.1016/j.jacr.2015.03.007. 25963225

[2] J. Slipsager , S. Glimberg , J. Søgaard , et al., “Quantifying the Financial Savings of Motion Correction in Brain MRI: A Model‐Based Estimate of the Costs Arising From Patient Head Motion and Potential Savings From Implementation of Motion Correction,” Journal of Magnetic Resonance Imaging 52, no. 3 (2020): 731–738, 10.1002/jmri.27112. 32144848

[3] F. Godenschweger , U. Kägebein , D. Stucht , et al., “Motion Correction in MRI of the Brain,” Physics in Medicine and Biology 61, no. 5 (2016): R32–R56, 10.1088/0031-9155/61/5/R32. 26864183 PMC4930872

[4] M. Zaitsev , J. Maclaren , and M. Herbst , “Motion Artifacts in MRI: A Complex Problem With Many Partial Solutions,” Journal of Magnetic Resonance Imaging 42, no. 4 (2015): 887–901, 10.1002/jmri.24850. 25630632 PMC4517972

[5] Y. Chang , Z. Li , G. Saju , H. Mao , and T. Liu , “Deep Learning‐Based Rigid Motion Correction for Magnetic Resonance Imaging: A Survey,” Meta‐Radiology 1, no. 1 (2023): 100001, 10.1016/j.metrad.2023.100001.

[6] V. Spieker , H. Eichhorn , K. Hammernik , et al., “Deep Learning for Retrospective Motion Correction in MRI: A Comprehensive Review,” IEEE Transactions on Medical Imaging 43, no. 2 (2024): 846–859, 10.1109/tmi.2023.3323215. 37831582

[7] D. Atkinson , D. Hill , P. Stoyle , P. Summers , and S. Keevil , “Automatic Correction of Motion Artifacts in Magnetic Resonance Images Using an Entropy Focus Criterion,” IEEE Transactions on Medical Imaging 16 (1997): 903–910, 10.1109/42.650886. 9533590

[8] A. Loktyushin , H. Nickisch , R. Pohmann , and B. Schölkopf , “Blind Retrospective Motion Correction of MR Images,” Magnetic Resonance in Medicine: Official Journal of the Society of Magnetic Resonance in Medicine/Society of Magnetic Resonance in Medicine 70 (2013): 1608–1618, 10.1002/mrm.24615. 23401078

[9] M. W. Haskell , S. F. Cauley , and L. L. Wald , “TArgeted Motion Estimation and Reduction (TAMER): Data Consistency Based Motion Mitigation for MRI Using a Reduced Model Joint Optimization,” IEEE Transactions on Medical Imaging 37, no. 5 (2018): 1253–1265, 10.1109/TMI.2018.2791482. 29727288 PMC6633918

[10] L. Cordero‐Grande , R. P. Teixeira , E. Hughes , J. Hutter , A. Price , and J. Hajnal , “Sensitivity Encoding for Aligned Multishot Magnetic Resonance Reconstruction,” IEEE Transactions on Computational Imaging 2 (2016): 266–280, 10.1109/TCI.2016.2557069.

[11] L. Cordero‐Grande , G. Ferrazzi , R. P. A. G. Teixeira , J. O'Muircheartaigh , A. N. Price , and J. V. Hajnal , “Motion‐Corrected MRI With DISORDER: Distributed and Incoherent Sample Orders for Reconstruction Deblurring Using Encoding Redundancy,” Magnetic Resonance in Medicine 84, no. 2 (2020): 713–726, 10.1002/mrm.28157. 31898832 PMC7392051

[12] M. W. Haskell , S. F. Cauley , B. Bilgic , et al., “Network Accelerated Motion Estimation and Reduction (NAMER): Convolutional Neural Network Guided Retrospective Motion Correction Using a Separable Motion Model,” Magnetic Resonance in Medicine 82, no. 4 (2019): 1452–1461, 10.1002/mrm.27771. 31045278 PMC6626557

[13] D. Polak , D. N. Splitthoff , B. Clifford , et al., “Scout Accelerated Motion Estimation and Reduction (SAMER),” Magnetic Resonance in Medicine 87, no. 1 (2022): 163–178, 10.1002/mrm.28971. 34390505 PMC8616778

[14] N. M. Singh , N. Dey , M. Hoffmann , et al., “Data Consistent Deep Rigid MRI Motion Correction,” Proceedings of Machine Learning Research 227 (2024): 368–381. 39415845 PMC11482239

[15] B. A. Duffy , L. Zhao , F. Sepehrband , et al., “Retrospective Motion Artifact Correction of Structural MRI Images Using Deep Learning Improves the Quality of Cortical Surface Reconstructions,” NeuroImage 230 (2021): 117756, 10.1016/j.neuroimage.2021.117756. 33460797 PMC8044025

[16] Y. Sun , L. Wang , G. Li , W. Lin , and L. Wang , “A Foundation Model for Enhancing Magnetic Resonance Images and Downstream Segmentation, Registration and Diagnostic Tasks,” Nature Biomedical Engineering 9, no. 4 (2025): 521–538, 10.1038/s41551-024-01283-7. PMC12360180 39638876

[17] O. Solomon , R. Patriat , H. Braun , et al., “Motion Robust Magnetic Resonance Imaging via Efficient Fourier Aggregation,” Medical Image Analysis 83 (2023): 102638, 10.1016/j.media.2022.102638. 36257133 PMC10291887

[18] M. Usman , S. Latif , M. Asim , B. D. Lee , and J. Qadir , “Retrospective Motion Correction in Multishot MRI Using Generative Adversarial Network,” Scientific Reports 10, no. 1 (2020): 4786, 10.1038/s41598-020-61705-9. 32179823 PMC7075875

[19] L. Beljaards , N. Pezzotti , C. Rao , M. Doneva , M. J. van Osch , and M. Staring , “AI‐Based Motion Artifact Severity Estimation in Undersampled MRI Allowing for Selection of Appropriate Reconstruction Models,” Medical Physics 51, no. 5 (2024): 3555–3565, 10.1002/mp.16918. 38167996

[20] J. G. Pipe , “Motion Correction With PROPELLER MRI: Application to Head Motion and Free‐Breathing Cardiac Imaging,” Magnetic Resonance in Medicine 42, no. 5 (1999): 963–969, 10.1002/(sici)1522-2594(199911)42:5963::aid-mrm173.0.co;2-l. 10542356

[21] S. Kecskemeti , A. Samsonov , J. Velikina , et al., “Robust Motion Correction Strategy for Structural MRI in Unsedated Children Demonstrated With Three‐Dimensional Radial MPnRAGE,” Radiology 289 (2018): 180180, 10.1148/radiol.2018180180. PMC6192848 30063192

[22] K. P. Pruessmann , M. Weiger , P. Börnert , and P. Boesiger , “Advances in Sensitivity Encoding With Arbitrary k‐Space Trajectories,” Magnetic Resonance in Medicine 46, no. 4 (2001): 638–651, 10.1002/mrm.1241. 11590639

[23] S. Guhaniyogi , M. L. Chu , H. C. Chang , A. W. Song , and C. Nk , “Motion Immune Diffusion Imaging Using Augmented MUSE for High‐Resolution Multi‐Shot EPI,” Magnetic Resonance in Medicine 75, no. 2 (2016): 639–652, 10.1002/mrm.25624. 25762216 PMC4567534

[24] M. R. Hestenes and E. Stiefel , “Methods of Conjugate Gradients for Solving Linear Systems,” Journal of Research of the National Bureau of Standards 49 (1952): 409–435.

[25] C. Metz , S. Klein , M. Schaap , T. Walsum , and W. Niessen , “Nonrigid Registration of Dynamic Medical Imaging Data Using *n*D+t B‐Splines and a Groupwise Optimization Approach,” Medical Image Analysis 15 (2010): 238–249, 10.1016/j.media.2010.10.003. 21075672

[26] S. Klein , M. Staring , K. Murphy , M. A. Viergever , and J. P. W. Pluim , “Elastix: A Toolbox for Intensity‐Based Medical Image Registration,” IEEE Transactions on Medical Imaging 29, no. 1 (2010): 196–205, 10.1109/TMI.2009.2035616. 19923044

[27] D. P. Shamonin , E. E. Bron , B. P. Lelieveldt , M. Smits , S. Klein , and M. Staring , “Fast Parallel Image Registration on CPU and GPU for Diagnostic Classification of Alzheimer's Disease,” Frontiers in Neuroinformatics 7 (2014): 50, 10.3389/fninf.2013.00050. 24474917 PMC3893567

[28] G. Wu , H. Jia , Q. Wang , and D. Shen , “Groupwise Registration With Sharp Mean,” Medical Image Computing and Computer‐Assisted Intervention 13, no. Pt 2 (2010): 570–577, 10.1007/978-3-642-15745-5_70. 20879361 PMC3018690

[29] A. Wolny , L. Cerrone , A. Vijayan , et al., “Accurate and Versatile 3D Segmentation of Plant Tissues at Cellular Resolution,” eLife 9 (2020): e57613, 10.7554/eLife.57613. 32723478 PMC7447435

[30] Ö. Çiçek , A. Abdulkadir , S. S. Lienkamp , T. Brox , and O. Ronneberger , “3D U‐Net: Learning Dense Volumetric Segmentation From Sparse Annotation,” Medical Image Computing and Computer‐Assisted Intervention (MICCAI) 2016 (2016): 424–432.

[31] Z. Wang , A. C. Bovik , H. R. Sheikh , and E. P. Simoncelli , “Image Quality Assessment: From Error Visibility to Structural Similarity,” IEEE Transactions on Image Processing 13, no. 4 (2004): 600–612. 15376593 10.1109/tip.2003.819861

[32] G. Zeng , Y. Guo , J. Zhan , et al., “A Review on Deep Learning MRI Reconstruction Without Fully Sampled k‐Space,” BMC Medical Imaging 21, no. 1 (2021): 195, 10.1186/s12880-021-00727-9. 34952572 PMC8710001

[33] N. Pezzotti , S. Yousefi , M. S. Elmahdy , et al., “An Adaptive Intelligence Algorithm for Undersampled Knee MRI Reconstruction,” IEEE Access 8 (2020): 204825–204838.

[34] M. Buehrer , K. P. Pruessmann , P. Boesiger , and S. Kozerke , “Array Compression for MRI With Large Coil Arrays,” Magnetic Resonance in Medicine 57, no. 6 (2007): 1131–1139, 10.1002/mrm.21237. 17534913

[35] H. Eichhorn , R. Frost , A. Kongsgaard , et al. “Evaluating the Performance of Markerless Prospective Motion Correction and Selective Reacquisition in a General Clinical Protocol for Brain MRI,” preprint, PsyArXiv, November 9, 2022, 10.31234/osf.io/vzh4g.

[36] M. Brant‐Zawadzki , G. D. Gillan , and W. R. Nitz , “MP RAGE: A Three‐Dimensional, T1‐Weighted, Gradient‐Echo Sequence‐Initial Experience in the Brain,” Radiology 182, no. 3 (1992): 769–775, 10.1148/radiology.182.3.1535892. 1535892

[37] O. Maier , S. H. Baete , A. Fyrdahl , et al., “CG‐SENSE Revisited: Results From the First ISMRM Reproducibility Challenge,” Magnetic Resonance in Medicine 85, no. 4 (2021): 1821–1839, 10.1002/mrm.28569. 33179826 PMC8201869

[38] J. Hamilton , D. Franson , and N. Seiberlich , “Recent Advances in Parallel Imaging for MRI,” Progress in Nuclear Magnetic Resonance Spectroscopy 101 (2017): 71–95, 10.1016/j.pnmrs.2017.04.002. 28844222 PMC5927614

