# Supplementary material for: DEEP‐DISORDER: Motion Correction in 3D MRI via Segment Reconstruction and Registration
Source: NMR Biomed. 2026 Apr 9;39(5):e70286. doi: 10.1002/nbm.70286 (PMC13066780; doi:10.1002/nbm.70286)
Supplement: Supplementary file 1 — AI4MRI_DEEPDISORDER_Appendix.pdf. [file NBM-39-e70286-s001.pdf]

10 | APPENDIX

Table 3 shows motion-corrected reconstruction quality on 6 retrospectively motion-corrupted scans with 2 spoiled gradient echo 3D-scans with different echo-times. The echo times of the acquisitions were 7.2 and 13.4 ms with a TR of 31 ms and a flip angle of 17°, making the first appear closer to the T1w scans that the model was trained on (which had an echo time of 4.6 ms), while the second adds a bit more T2*-contrast into the images. The scans had an acquisition matrix of around size $368 \times 366 \times 70$ with a slice thickness of 2 mm and 1 mm spacing between slices. We center-padded the isotropic reconstructed k-space of each scan to obtain data with a uniform size of 384^3 voxels. Motion estimation was performed with DEEP-DISORDER on center-cropped data with size 192^3, i.e. on a lower resolution, while the final reconstructions were performed on size 384^3 data. The table shows the performance of DEEP-DISORDER using the segment-reconstruction model that was trained on T1w data exclusively, and a version that was fine-tuned on 21×2 spoiled gradient echo training scans.

The results demonstrate that the model performed well on the first echo time even without fine-tuning, but performed worse for the second echo time that is further from T1w data and has a less pronounced skull. Fine-tuning the T1-model on a limited number of spoiled gradient echo scans re-enabled accurate motion correction, restoring reconstruction performance even for the scans at 13.4 ms echo time. Figure 5 shows example reconstructions. It can be seen that DEEP-DISORDER can accurately correct for motion on higher-resolution data, even when the motion estimation is run on a lower resolution.

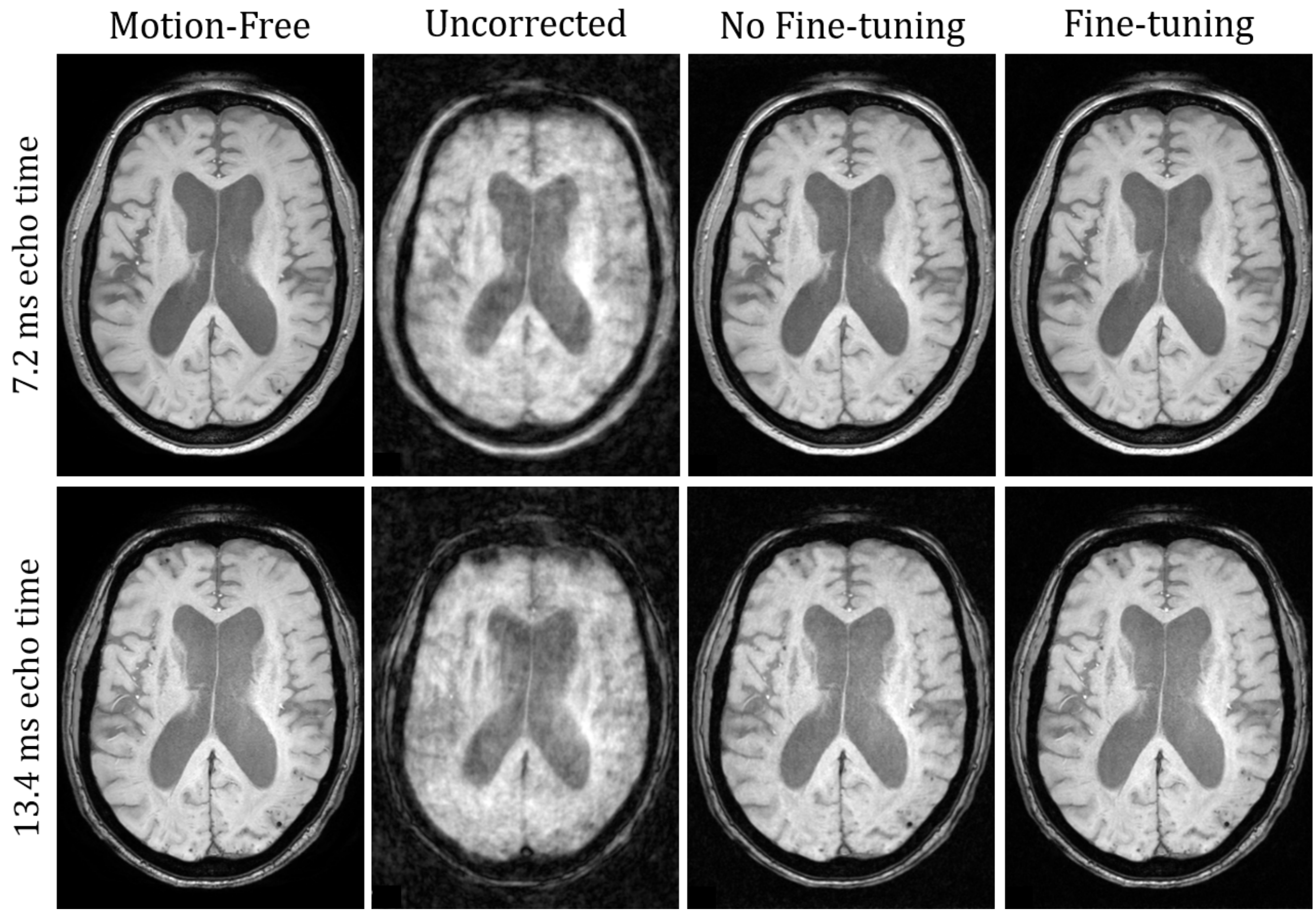

FIGURE 5 Motion-corrected reconstructions by DEEP-DISORDER of retrospectively motion-corrupted multi-echo spoiled gradient echo data with an echo time of 7.2 ms (top) and 13.4 ms (bottom) without and with fine-tuning.

Fine-tuning strategy	7.2 ms echo time		13.4 ms echo time	
	SSIM	PSNR	SSIM	PSNR
Uncorrected	0.826 ± 0.015	30.88 ± 1.28	0.805 ± 0.015	32.35 ± 1.06
No fine-tuning	0.988 ± 0.002	45.57 ± 1.35	0.967 ± 0.013	43.54 ± 2.60
Fine-tuning	0.992 ± 0.001	47.50 ± 1.17	0.989 ± 0.003	48.49 ± 1.59

TABLE 3 DEEP-DISORDER motion-corrected reconstruction quality ($\mu \pm \sigma$) on retrospectively motion-corrupted multi-echo spoiled gradient echo data with an echo time of 7.2 ms (left) and 13.4 ms (right).
